# Insights into One-Dimensional Thermoelectric Materials: A Concise Review of Nanowires and Nanotubes

**DOI:** 10.3390/nano14151272

**Published:** 2024-07-29

**Authors:** Giovanna Latronico, Hossein Asnaashari Eivari, Paolo Mele, Mohammad Hussein Naseef Assadi

**Affiliations:** 1 National Research Council of Italy Institute of Condensed Matter Chemistry and Technologies for Energy (CNR-ICMATE), Via G. Previati 1/E, 23900 Lecco, Italy; 2 Physics Department, Faculty of Science, University of Zabol, Zabol 98613-53856, Iran; 3 College of Engineering, Shibaura Institute of Technology, Omiya Campus, 307 Fukasaku, Minuma-ku, Saitama City 337-8570, Japan; 4 RIKEN Center for Emergent Matter Science (CEMS), Wako 351-0198, Japan; 5 Chemistry Department, Faculty of Engineering and Natural Sciences, Istinye University, Sarıyer, Istanbul 34396, Türkiye

**Keywords:** computational materials science, thermal conductivity, 1D thermoelectric nanomaterials, figure of merit, nanowire, carbon nanotubes, flexibility

## Abstract

This brief review covers the thermoelectric properties of one-dimensional materials, such as nanowires and nanotubes. The highly localised peaks of the electronic density of states near the Fermi levels of these nanostructured materials improve the Seebeck coefficient. Moreover, quantum confinement leads to discrete energy levels and a modified density of states, potentially enhancing electrical conductivity. These electronic effects, coupled with the dominance of Umklapp phonon scattering, which reduces thermal conductivity in one-dimensional materials, can achieve unprecedented thermoelectric efficiency not seen in two-dimensional or bulk materials. Notable advancements include carbon and silicon nanotubes and Bi_3_Te_2_, Bi, ZnO, SiC, and Si_1−*x*_Ge*_x_* nanowires with significantly reduced thermal conductivity and increased *ZT*. In all these nanowires and nanotubes, efficiency is explored as a function of the diameter. Among these nanomaterials, carbon nanotubes offer mechanical flexibility and improved thermoelectric performance. Although carbon nanotubes theoretically have high thermal conductivity, the improvement of their Seebeck coefficient due to their low-dimensional structure can compensate for it. Regarding flexibility, economic criteria, ease of fabrication, and weight, carbon nanotubes could be a promising candidate for thermoelectric power generation.

## 1. Introduction

When the dimensions of a material decrease, due to quantum confinement, its properties change significantly. Accordingly, the focus of materials science over the past few decades has been on how material properties can be manipulated by controlling dimensionality, as the potential applications are endless. Some possible applications include magnetic [1,2], optical [3,4], electronic [5,6], bioactive [7,8], mechanical [9,10], and thermoelectric (TE) [11,12] properties, of which, TE properties are being focused on in this minireview. Other instances encompass photocatalytic hydrogen production [13], artificial ligaments [14], printable mesoscopic solar cells [15], and flexible biomimetic e-whiskers [16].

Thermoelectricity is a property of materials that causes the conversion of a temperature difference into electricity and vice versa, thanks to two effects, respectively, named the Seebeck and Peltier effects. The first is the basis of thermoelectric generators (TEGs), which are simple and robust devices with no moving parts, making them perfect candidates for energy harvesting and powering wireless devices. In the current energy and pollution crisis and the exponential increase in the number of off-grid IoT devices, thermoelectricity could be one of the critical technologies in the future [17]. The Peltier effect has applications in silent cooling, where the current flowing in the device generates a temperature difference between the two sides of the module.

The thermoelectric figure of merit, *ZT*, is a dimensionless measure of the efficiency of a thermoelectric material; the higher its value, the higher the efficiency of the material. *ZT* is defined as:

*ZT* = (*S*^2^*σT*)/(*κ_e_* + *κ_l_*), (1)

where *S* is the Seebeck coefficient, *σ* is the electrical conductivity, *κ_e_* and *κ_l_* are electron thermal conductivity and phonon thermal conductivity, and *T* is the absolute temperature [18].

The primary current limitation on the large-scale application of this technology is its low efficiency. The arduous challenge lies in combining in the same solid material the electronic properties of a crystal and the thermal ones of glass, following the phonon glass electron crystal (PGEC) concept, decoupling the numerator and the denominator of Equation (1). The Wiedemann–Franz law puts constraints on the dependence of electrical conductivity and electron thermal conductivity:

*κ_e_* = *L*_0_*σT*, (2)

where *L*_0_ is the Lorenz number (2.45 × 10^−8^ W S^−1^ K^−2^). Consequently, it is impossible to independently manipulate the electronic contributions *σ* and *κ_e_* arbitrarily. An alternative route to increase *ZT* through a reduction in thermal conductivity is to work on the phonon component *κ_l_*, which can be mathematically expressed as:

*κ_l_* = ⅓*C_v_vΛ*, (3)

where *C_v_* is the specific heat at constant volume, *v* is the sound velocity, and *Λ* is the phonon mean free path. Since phonon propagation is highly sensitive to surface scattering via reduced *Λ*, nanoengineering 1D materials presents an unprecedented opportunity to improve thermoelectric efficiency.

Significant efforts are underway to improve TEGs. In this framework, new materials and new dopants are of great interest, but dimensionality has played a considerable role in the recent progress in the field [19,20]. Compared to bulk materials, significant improvements in the thermoelectric figure of merit, *ZT*, have been recently reported in nanostructures. The advantage of low dimensionality can be explained by the reduction in *κ_l_* and the improvement in the device’s power factor, *S*^2^*σ*. For thermoelectricity, using low-dimensional materials introduces additional freedom through the length scale. This unique characteristic enables the deliberate manipulation of the materials’ electronic and thermal properties, resulting in enhanced thermoelectric performance.

Reduced dimensionality leads to electron and phonon quantum confinement, resulting in discrete energy levels and a modified density of states (DOS) [21,22]. Electronically, this confinement can enhance electrical conductivity, depending on the alignment of energy levels and carrier-scattering mechanisms [23]. In nanowires and nanotubes, carriers are confined to move along a single axis, which can increase carrier mobility by reducing scattering rates, provided the structure is chemically pure and defect free. However, surface scattering and edge effects can significantly impact electrical conductivity, causing considerable anisotropy [24]. The enhancement of the Seebeck coefficient in low-dimensional materials can be attributed to the localisation of the DOS in one-dimensional (1D) sub-bands, expressed as *S*(*E*) ∝ [δ(DOS)/*δE*]/[DOS] [25]. For phonons, Umklapp scattering, a process where phonon momentum is not conserved within the first Brillouin zone, becomes more significant. This process impedes the involved phonon’s heat-carrying capacity, reducing thermal conductivity [26]—albeit with caveats [27]. Additionally, in nanowires and nanotubes, phonons frequently encounter the surfaces of the wires or tubes, leading to significant surface scattering. These scatterings reduce the mean free path of phonons, further lowering thermal conductivity [28]. Compared to bulk materials or two-dimensional nanomaterials, a significant advantage of one-dimensional structures is the flexibility of the resulting devices, which can be bent without delamination or cracking [29]. However, it is important to note that 2D thermoelectric materials have unique niche applications due to their relatively easy synthesis via exfoliation from layered bulk materials [30,31]. Undoubtedly, one-dimensional materials still offer many possibilities for engineering highly efficient and practical thermoelectric devices.

So far, researchers have reported several types of one-dimensional nanostructures that have shown improved efficiency in thermoelectric applications. These include superlattice structures [32], silicon nanowires (NWs) [33], both carbon and non-carbon nanotubes (NTs) [34], and nanocomposite structures [35]. Numerous publications have explored this subject, and interested readers can find more comprehensive information in these excellent reviews [34,36,37,38]. This article provides a brief overview of the performance of standard classes of one-dimensional thermoelectric materials, explicitly focusing on the computational aspects of thermal conductivity, which are arguably the most challenging to manage due to numerical complexity [11,39].

## 2. Non-Flexible Inorganic 1D Thermoelectric Materials

**Figure 1:**
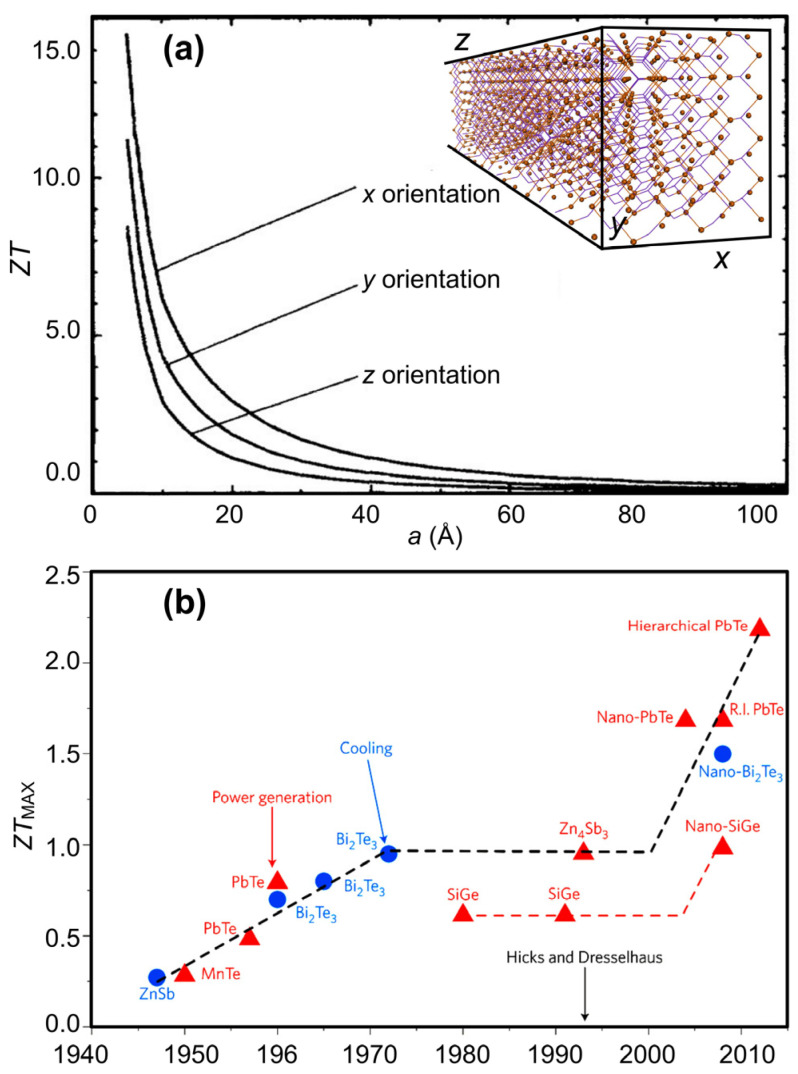
(**a**) Theoretical *ZT* values vs. diameter for an anisotropic conducting nanowire of a square cross section fabricated along the lattice vector’s *x*, *y*, and *z* directions. (**b**) The progression in *ZT* improvement through the years. Adapted with permission from Refs. [37,40]. Copyrights 1993 American Physical Society and 2013 Springer Nature.

In 1993, Hicks and Dresselhaus [40], using analytical and statistical calculations, predicted that quantum effects could provide a new way to design thermoelectric materials. As indicated in Figure 1a, in the case of nanowires, the calculated *ZT* increases significantly with decreasing wire diameter below the thermal de Broglie wavelength of the carriers. This increase is because of a change in the density of states. In addition, in 1D structures, electron movements are confined to a single dimension parallel to the nanowire. Such confinement leads to no scattering off the surface. Therefore, the mobility of carriers in the nanowire direction remains unchanged.

Furthermore, the increased phonon scattering from the surface of a thin wire leads to a reduction in the structure’s thermal conductivity and, hence, an increase in *ZT*. Accordingly, Hicks and Dresselhaus predicted a *ZT* as high as 14 for a Bi_2_Te_3_ nanowire of 5 Å diameter. The outcomes of these theoretical predictions definitely demonstrate the immense potential of quantum nanowire structures as thermoelectric nanomaterials with a high *ZT*. As depicted in Figure 1b, the groundbreaking works by Hicks and Dresselhaus—credited with seminal papers exploring the figure of merit for confined spaces in two-dimensional [41] and one-dimensional [40] systems—ushered in a new era of thermoelectric research. These publications marked a significant milestone in the field, propelling the investigation of TE materials towards the realm of nanoengineering, subsequently leading to remarkable advancements in their thermoelectric figure of merit [11,12].

**Figure 2:**
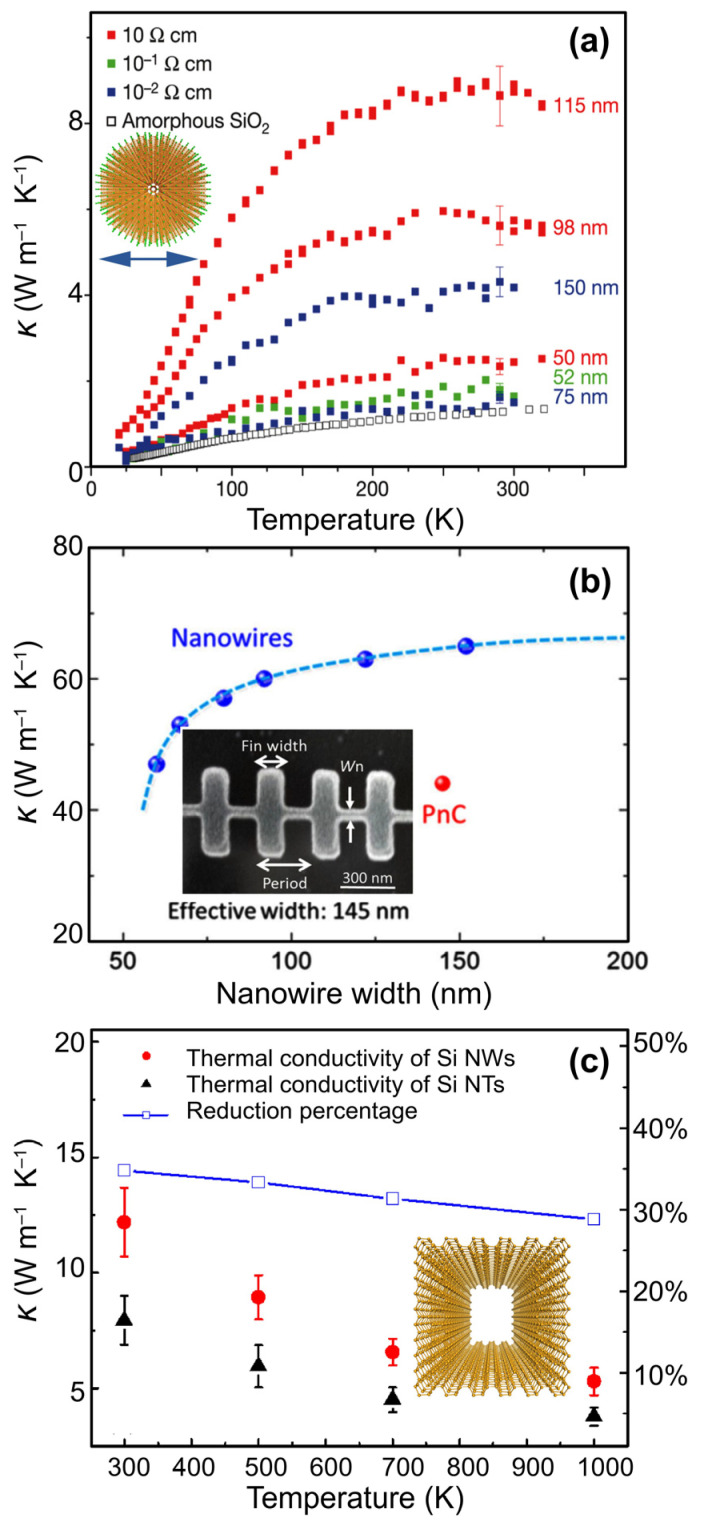
(**a**) The dependence of thermal conductivity on temperature for Si nanowires with different diameters scratched from wafers of different resistivities. The curve with open squares is the thermal conductivity of bulk amorphous Si. (**b**) The thermal conductivity of a fishbone-like Si nanostructure compared to that of similar-width nanowires. (**c**) The dependence of thermal conductivity on temperature for Si nanowires (Si NWs) and Si nanotubes (Si NTs). The cross-section areas for Si NTs and Si NWs are 7.30 nm^2^ and 7.37 nm^2^, respectively. In (**c**), the right-axis labels show the reduction in *κ* in nanotubes compared to that in same-width nanowires. The insets in (**a**,**c**) show the nanowire and nanotube structures. Adapted with permission from Refs. [33,42,44]. Copyrights 2008 and 2014 Nature Springer and 2010 American Chemical Society.

Hochbaum et al. reported the synthesis of large-area, wafer-scale Si nanowires [33]. The result of the thermal conductivity measurement in their work is shown in Figure 2a. The thermal conductivity of bulk Si was about 150 W m^−1^ K^−1^. However, nanowires with diameters of ~50 nm exhibited a much smaller thermal conductivity of ~4 W m^−1^ K^−1^, which approached the amorphous limit for Si. Additionally, the Seebeck coefficient and the electrical conductivity of the wires remained comparable to those of doped bulk Si. As a result, the nanowires had a *ZT* of 0.6. The significant reduction in thermal conductivity was attributed to nanowires’ surface roughness, which strongly screens a broad spectrum of phonons and alters the phonon transmission through the confined structures. Silicon is environmentally friendly and abundant. Further, the current silicon-based microelectronic industry is highly developed. Consequently, the significant *ZT* enhancement observed in Si NWs and their numerous inherent advantages have propelled researchers to seriously consider Si nanostructures as up-and-coming candidates for thermoelectric applications.

Nomura et al. fabricated fishbone-type silicon phononic crystal (PnC) nanostructures via electron beam lithography and measured the thermal conductivity via micro-time-domain thermoreflectance [42]. Their experiment showed that the PnC had lower thermal conductivity than a nanowire with a corresponding width (Figure 2b). More specifically, for an effective width of 145 nm, the thermal conductivity was lowered from around 65 W m^−1^ K^−1^ to ~45 W m^−1^ K^−1^. Nonetheless, comparing the theoretical band engineering analysis and the thermal conductivity measurements on several fishbone-like PnC nanostructures with different fin-width-to-period ratios raised some discrepancies, which could be explained by the increase in the travelling distance of the phonons within the fin part of the nanostructure.

Zhang et al. [43] were able to fabricate through a metal-assisted chemical etching method some high-density and large-area, vertically aligned porous silicon nanowire arrays (SiNWAs) on the two sides of silicon substrates, called sandwich structured composites (SSCs), with control over porosity and length. They investigated the SSCs’ thermoelectric properties to obtain the *ZT* values of the corresponding SiNWAs at room temperature. They observed that *ZT* increases, together with the length and porosity of the structures (up to 547 m^2^ g^−1^). The peak value of the figure of merit was 0.493 for a large-porosity SiNWA, which is 77 times higher than that of bulk silicon (0.0064), thanks also to a remarkable Seebeck coefficient, equal to 513 µV K^−1^.

By introducing a small hole at the centre of the Si NW, i.e., constructing a silicon nanotube (NT) structure, Chen et al. [44] demonstrated that the room-temperature thermal conductivity of the structure could be reduced by 35%, as shown in Figure 2c. Holey Si was fabricated by dry-etching silicon on an insulator wafer. In a similar work, the lattice thermal conductivity of holey Si was reported to be close to that of amorphous SiO_2_ at room temperature, and its *ZT* value was reported to be 0.4 [45]. However, competitive thermoelectric performance in 1D Si nanostructures still required a further reduction in thermal conductivity. Subsequently, a follow-up experimental study [46] reported an excellent power factor and low thermal conductivity of 1.8 W m^−1^ K^−1^, close to the amorphous limit, for silicon nanowires. The work presented a silicon-based thermoelectric generator made of a collection of highly *p*-doped silicon nanowires, remarkably enhancing the electrical power density. The authors argued that the generator is suitable for applications exploiting small Δ*T* values.

Apart from Si-based nanowires, in a recent experimental work [47], a nanostructure-integrated chip composed of CdSSe nanowires was reported to be an excellent photo-thermoelectric generator. The Seebeck coefficient and the thermoelectric output voltage of the chip were reported to be −152.4 Μv K^−1^ and 10.8 mV, respectively. Moreover, the authors reported a significant elevation in the output voltage, up to 45 mV, upon illumination by white light. The elevation of the voltage was attributed to the high concentration of photo-generated charge carriers.

Alongside these experimental breakthroughs, computational investigations have regularly provided new insights into one-dimensional thermoelectricity. Neophytou et al. [48] conducted a simplified tight-binding density functional investigation to study thermoelectricity in Si NWs using 20 orbitals for Si atoms and accounting for the spin–orbit coupling. Their models consisted of Si NW of different diameters constructed from cylindrical cuts through Si’s zincblende lattice. Their results demonstrated that for Si NWs with diameters below 7 nm, the power factor and *ZT* values can be enhanced by up to 100% compared to bulk values. Another theoretical investigation by Sansoz [49], with non-equilibrium molecular dynamics calculations with the Stillinger–Weber potential [50], showed that nanoengineering the Si NW’s outer surface can substantially reduce the lattice thermal conductivity. According to the author’s prediction, the smooth-surfaced and circular Si NW had a thermal conductivity of 36 W m^−1^ K^−1^. In contrast, the Si NW with a saw-toothed surface with (111)/(100) facet terminations had a thermal conductivity of 16 W m^−1^ K^−1^, which is 10 times smaller than the prediction for bulk Si. Moreover, using Monte Carlo simulations for phonon transport within the diffuse and ballistic limits, Jean et al. [51] studied heat conduction in Si nanowires that were 2 μm long and 115 nm wide. They particularly investigated the role of constrictions, a pinch-like narrowing in the middle of Si NWs. It was clear that the thermal conductivity of the NW can be lowered and modulated by up to 90% through a fine adjustment of the steepness of the constrictions, its diameter ratio to the nanowire’s width, its length, and other parameters. The minimum *κ* was predicted to be less than 10 µW m^−1^ K^−1^.

**Figure 3:**
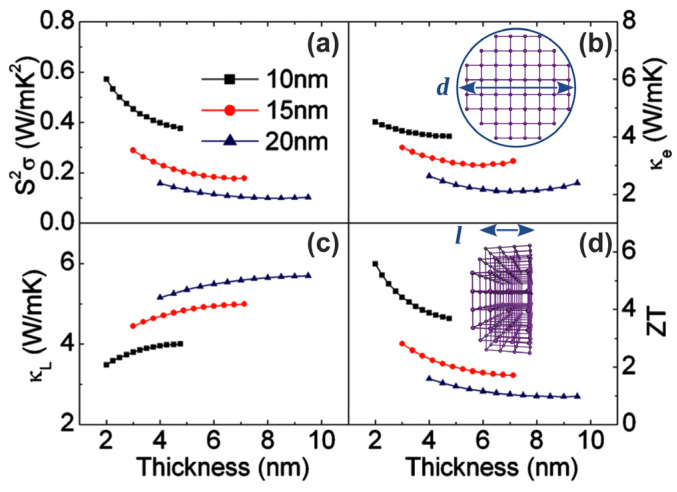
Variations in the calculated optimum power factor (**a**), electron thermal conductivity (**b**), lattice thermal conductivity (**c**), and optimum thermoelectric *ZT* (**d**) with thickness for *n*-type Bi nanotubes with different diameters oriented along the trigonal direction at 77 K. Adapted with permission from Ref. [53]. Copyright 2010 American Institute of Physics.

Additionally, the thermoelectric properties of one-dimensional Bi nanowires and nanotubes were shown to depend on diameter critically. Theoretical investigations using a semiclassical transport model indicated that the *ZT* of *n*-type Bi NWs with a diameter of 5 nm can exceed 6 at 77 K [52]. Moreover, by combining the effective mass envelope wave function and the Boltzmann transport equation, the theoretical results in Figure 3 show the effect of the diameter and thickness of *n*-type Bi nanotubes on their power factor, electron thermal conductivity, lattice thermal conductivity, and maximum *ZT* at 77 K. As a final result, panel d indicates that when the thickness and diameter of Bi NTs decrease to 2 nm and 10 nm, the value of their *ZT* can increase to 6 [53].

Further theoretical calculations using equilibrium molecular dynamics and the Green–Kubo formula [54,55] showed that compared to bulk zinc oxide, the *ZT* value of ZnO nanowires increases by 30 times, reaching ~0.06, when the diameter of the wire decreases to 8 Å [56]. All electron density functional calculations with generalised gradient approximation [57] and the Boltzmann transport model on *n*-type silicon–germanium (Si_1−*x*_Ge*_x_*) nanowires ((110) direction with 2.3 nm^2^ rectangular cross section) predicted that the *ZT* value can be increased by 4.3 times over that of Si of the same dimensionality [58]. In corroboration with this prediction regarding Si-based nanowires, an experimental investigation showed that the figure of merit of *β*-SiC nanowires (diameters of 60~100 nm) is about 120 times higher than the maximum *ZT* value of bulk SiC, reaching values up to 0.12 [59].

## 3. Flexible Thermoelectric Devices with 1D Carbon Nanotubes

**Figure 4:**
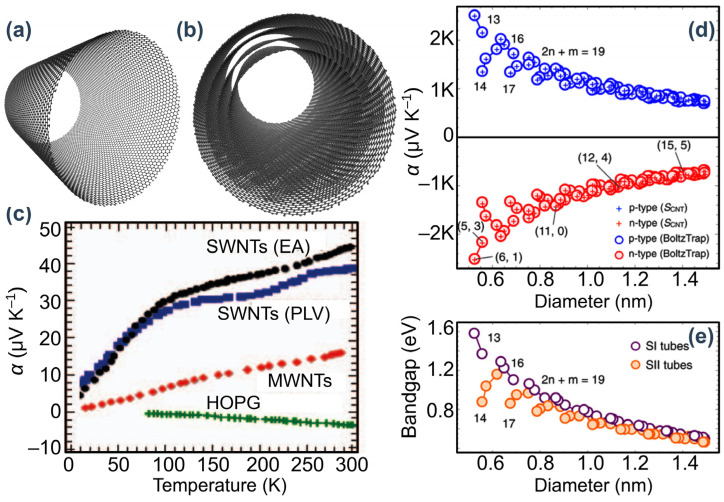
(**a**,**b**) Schematic representation of single-walled and multi (four)-walled carbon nanotubes. (**c**) Dependence of the Seebeck coefficient *α* on temperature for air-saturated single-walled nanotubes (SWNTs), multi-walled nanotubes (MWNTs), and highly oriented pyrolytic graphite (HOPG). EA and PLV refer to synthesis methods, i.e., electric arc and pulsed laser vaporisation. (**d**) Optimum thermopower (Seebeck coefficient *α*) values vs. SWNT diameter for s-SWNTs at room temperature. (**e**) The Kataura plot shows the family pattern of the SWNT bandgap vs. diameter. Adapted with permission from Refs. [62,65]. Copyrights 2006 Springer and 2015 American Physical Society.

Due to inherent brittleness and rigidity, conventional bulk TE materials, which are based on inorganic semiconductors, cannot establish contact with the curved surfaces of heat sources and sinks well enough. This rigidity imposes limitations on their application in modern electronics. So, it is desired to fabricate flexible TE materials with high thermoelectric performance. Carbon nanotubes (CNTs), shown in Figure 4a,b, have some potential utility in overcoming this limitation. CNTs are attractive because they are flexible, and their source materials are abundant and nontoxic [60]. They can also be fabricated using high-throughput solution-phase synthesis techniques, and they have high specific energy (i.e., W g^−1^) enabled by their low mass [61]. Also, the thermal properties of CNT arrays are tuneable. There are detailed reviews of CNT TE materials, including theoretical and experimental efforts [36], so the coverage here is concise.

Commonly, three methods are used to synthesise CNTs [62]: electric arc discharge, chemical vapour deposition (CVD), and pulsed laser vaporisation. Figure 4c shows a general comparison between Seebeck coefficients of highly oriented pyrolytic graphite (HOPG), multi-walled CNTs (MWCNTs), and single-walled CNTs (SWCNTs). All samples were subjected to ambient atmosphere and light for a long time so they could be considered sufficiently oxygen doped. The figure shows that SWCNTs and MWCNTs have room-temperature Seebeck coefficients of approximately 45 µV K^−1^ and 17 µV K^−1^, respectively. These values are much higher than that of HOPG. Figure 4c indicates the enhancement of the Seebeck coefficient with a reduction in the size of the CNTs. The sign of the Seebeck coefficient of SWCNTs and MWCNTs is reported to be positive, indicating that oxygen-doped nanotubes are *p*-type.

Although CNTs have high thermal conductivity [63], which is unacceptable for thermoelectric applications, a computational study demonstrated that a semiconducting SWCNT can be optimised to make its thermoelectric performance competitive [64]. The study revealed that *p*-type SWCNTs show higher power factors originating from their more significant electrical conductivity, which was attributed to a relatively larger DOS around the valence band maximum and longer hole relaxation times. It also showed that the *ZT* value of the CNT at elevated temperatures can be remarkably enhanced to 1.9 by a reduction in its thermal conductivity using isotopic substitution and hydrogen sorption. Consequently, superior electronic transport properties of earth-abundant and environmentally friendly CNTs, coupled with an effective reduction in their thermal conductivity, could guarantee viable thermoelectric performance. Moreover, based on the electronic structure, the SWCNT can be either metallic (m-SWCNT) or semiconductor (s-SWCNT). Statistical reports indicate that one-third of all possible SWCNTs show metallic behaviour, while two-thirds behave as semiconductors. This ratio is maintained in almost all SWCNT synthesis procedures. Efforts have been made to develop methods to separate as-prepared SWCNT samples based on diameter, electronic structure, and chirality [36].

The thermopower dependence on the electronic structure and diameter of CNTs is depicted in Figure 4d. The figure is a part of the results of theoretical calculations carried out by Hung et al. [65] for s-SWCNTs using a method in which the Boltzmann transport formalism was combined with an extended tight-binding model. The thermopower trend (Figure 4d) demonstrated a strong relationship between enhanced thermopower and reduced nanotube diameter. Benchmarking α values against the bandgaps reported in Figure 4e indicated that the thermopower of a given s-SWNT is also related to its bandgap. Accordingly, for a tube with a small diameter, the thermopower could exceed 2000 μV K^−1^ at room temperature. This value is about 10 times higher than that of commonly used thermoelectric materials. As reported by the authors, the large thermopower of nanotubes is related to their one-dimensionality and the large bandgaps of s-SWNTs with small diameters. More recently, Taborowska et al. [66] experimentally demonstrated the potential application of carbon nanotubes (CNTs) for thermoelectric purposes through substantial molecular doping and the inclusion of ZnO nanowires. This treatment enhanced the electrical conductivity of pure single-walled carbon nanotube (SWCNT) arrays by a factor of 3. Additionally, the power factor improved from 34.9 µW m^−1^ K^−2^ for unmodified SWCNTs to 42.91 µW m^−1^ K^−2^ for CNTs treated with molecular doping and ZnO nanowire inclusion.

**Figure 5:**
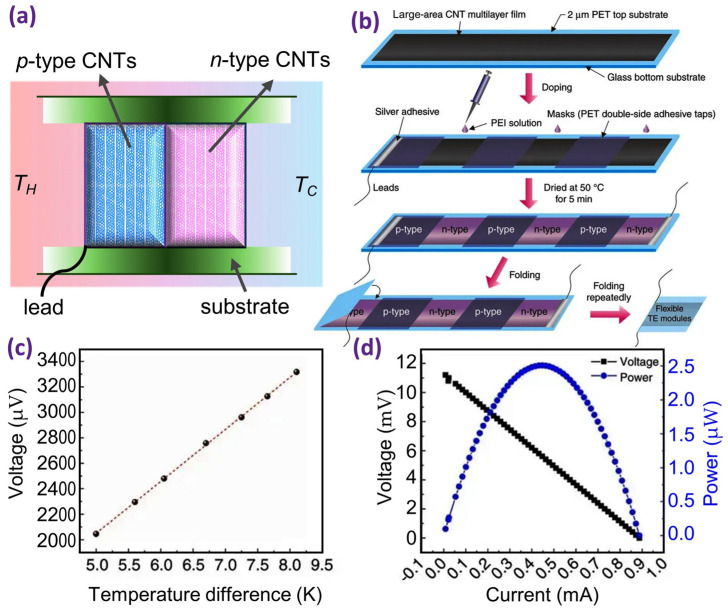
(**a**) A thermoelectric module made of alternating *p*- and *n*-type doped CNTs operating between hot (*T_H_*) and cold (*T_C_*) reservoirs. (**b**) Schematics of the fabrication process for sequenced *p*- and *n*-type TE components based on large-area continuously synthesised CNT films and localised doping technology. (**c**) The voltage generated between the two ends of the module in different steady-temperature differences. (**d**) The voltage–current curve and the power–current curve of the module at a hot-side temperature of 330 K and a temperature difference of 27.5 K. Adapted from Ref. [67]. Copyright 2017 the authors (CC BY 4.0).

Experimentally, Zhou et al. reported a straightforward method for fabricating a compact and flexible thermoelectric (TE) device, shown in Figure 5a, leveraging large-area continuous synthesis of CNT films and localised doping technology [67]. Figure 5b shows the schematics of the fabrication process. The prepared module showed low internal resistivity and did not require gold or silver top electrode deposition on each TE device or metallic interconnections between *n*- and *p*-type legs, further simplifying the assembly process. This technique effectively avoids design complications related to contact resistance and can be easily scaled up for large-scale production. The output voltages of the module at various steady temperature differences are depicted in Figure 5c. Figure 5d shows the voltage–current and power–current curves of the module under the condition in which the hot-side temperature is *T_H_* = 330 K and the steady-state temperature difference is Δ*T* = 27.5 K. At the mentioned temperatures, the power density of the module was estimated to be 167 µWcm^−2^. In this instance, because of the relatively high thermal conductivity, the CNT-based TE modules seem suitable for working at low-temperature differences. Furthermore, in a more recent experimental work [68], the fabrication of a flexible thermoelectric device based on a Bi_2_Te_3_–carbon nanotube hybrid was reported. The hybrid structure shows good flexibility under bending. Thermoelectrically, it exhibits a *ZT* value of about 0.23 at 330 K and a maximum output power density of about 0.93 mWcm^−2^ under a steady temperature difference of 25 K at room temperature.

**Figure 6:**
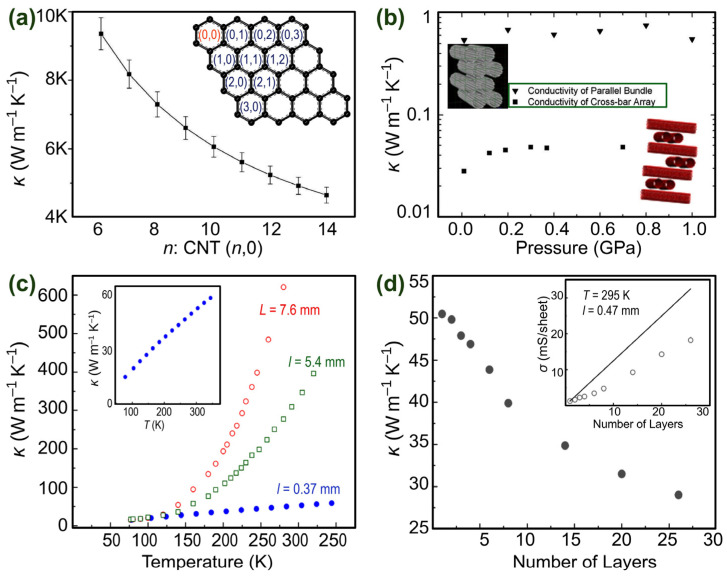
(**a**) Theoretical calculations showing the relationship between thermal conductivity and CNT diameter parameterised by increasing the first component of the (*n*, 0) vector. The inset shows how folding graphene produces a (*n*, *m*) CNT. (**b**) Pressure dependence of cross-plane thermal conductivity of single-walled (10, 10) carbon nanotubes arranged in a parallel bundle structure, shown in the upper-left inset, and a crossbar array, shown in the lower-right inset. (**c**) Measurement of thermal conductivity as a function of temperature along mono-walled carbon nanotube sheets of different lengths: 7.6 mm (open circles), 5.4 mm (open squares), and 0.37 mm (solid circles). (**d**) Thermal conductivity of mono-walled carbon nanotube sheets versus several superimposed layers with parallel alignment. Adapted with permission from Refs. [75,76,80]. Copyrights 2004 American Physical Society, 2007 Elsevier, and 2012 American Institute of Physics.

Notably, depending on their structure form (individual, films, bundled, bucky papers, etc.) and method of synthesis, CNTs’ thermal conductivity varies from the nearly thermal insulation, with a thermal conductivity of about 0.1 W m^−1^ K^−1^, to such high values as 6600 W m^−1^ K^−1^ [36,69]. This variability is partly due to thermal energy transport through a CNT being predominantly governed by the phonon conduction mechanism [70,71]. Even in metallic CNTs, heat transport is dominated by the collective vibration of atoms [72]. Subsequently, topological defects in a CNT, such as simple or Stone–Wales-type vacancies and extraneous impurities (other forms of carbon, residual catalyst, etc.), scatter the phonons and decrease their mean free path. In addition, CNT–CNT coupling in bundled samples can decrease thermal conductivity by about an order of magnitude compared to isolated CNTs [73]. Generally, in a stand-alone CNT, thermal conductivity increases with the CNT length up to the mean free path of phonons, which is about ~500 nm for MWCNTs and SWCNTs [74]. Furthermore, thermal conductivity is also predicted to decrease with increasing CNT diameter. In this regard, using the first-order perturbation theory with constant relaxation time and the three-phonon Umklapp process, Cao et al. found a nearly inverse power relationship between the CNT’s diameter and its thermal conductivity [75] (Figure 6a).

Evans et al. [76] conducted non-equilibrium molecular dynamics simulations using Tersoff and Lennard Jones potentials, accounting for both covalent and dispersion interaction, for two types of (10, 10) CNT bundles, namely crossbar and parallel arrays, as shown in Figure 6b. They found that at atmospheric pressure, crossbar and parallel arrays have a considerably low out-of-plane thermal conductivity of 0.028 and 0.55 W m^−1^ K^−1^, respectively. These *κ* values are orders of magnitude lower than the axial thermal conductivity of isolated CNTs (observed to be 3000~6000 W m^−1^ K^−1^ [74]). When comparing the two bundles, one should note that because of the unified orientation and much larger contact area among the CNTs, the thermal conductivity of the parallel array at low pressure is still a few times larger than that of the crossbar array.

Reverse non-equilibrium molecular dynamics (MD) simulation based on the AIREBO potential [77] conducted by Boroushak et al. [78] showed a reduction in thermal conductivity when the outer sides of single-walled (10, 10) and double-walled (5, 5)@(10, 10) CNTs were functionalised with polyethylene (PE) groups. The calculations showed that thermal conductivity decreases with increasing weight percentage of the functional group, i.e., a ~50 decrease in *κ* for ~25% of the polymer weight ratio. According to these results, the thermal conductivity of the SWCNT is more sensitive to the change in the weight percentage of functional groups. Moreover, using atomistic Green’s function calculations, Chalopin et al. [79] found that the phonon thermal conductivity of densely compacted pellets composed of almost 1 µm long carbon nanotubes has an upper bound of about 5 W m^−1^ K^−1^, which is about a thousand times lower than that of an individual CNT or graphene. They also predicted that the upper *κ* bound is not dependent on the nanotube diameter at room temperature.

An experimental study on sheets of different lengths of MWCNTs was reported by Aliev et al. [80]. As shown in Figure 6c, in the temperature range of 75~150 K, the length of MWCNT sheets does not influence the thermal conductivity value in a significant way (Figure 6c). However, above 150 K, longer samples had higher thermal conductivities. They also found that at *T* = 295 K, the thermal conductivity value along the films with a length of about 0.37 mm is 50 W m^−1^ K^−1^, while across the films, the conductivity value is 2.1 W m^−1^ K^−1^. Figure 6d shows that in multilayer systems, thermal conductivity decreases with increasing numbers of layers. The authors attributed the observed low thermal conductivity value to the respective nanotubes’ internal defects and the boundaries’ phonon scattering in bundles forming the basis of MWCNT sheets.

To summarise, comparing the theoretical and experimental findings discussed before, it becomes evident that computational predictions can confidently be considered an upper bound for thermal conductivity. For example, Berber et al. conducted molecular dynamics simulations using the Tersoff potential to calculate the thermal conductivity of isolated (10, 10) CNTs, resulting in *κ* = 6600 W m^−1^ K^−1^ [81]. However, most experimental reports find *κ* to be significantly lower. This discrepancy arises from the idealised structural models used in simulations. In reality, most synthetic methods, especially top-down approaches, produce materials with numerous defects [82,83]. These defects introduce various phonon-scattering mechanisms, reducing thermal conductivity. Notably, when simulations account for these structural complexities, the agreement between theory and experiment improves [76,78].

## 4. Outlook and Perspective

**Figure 7:**
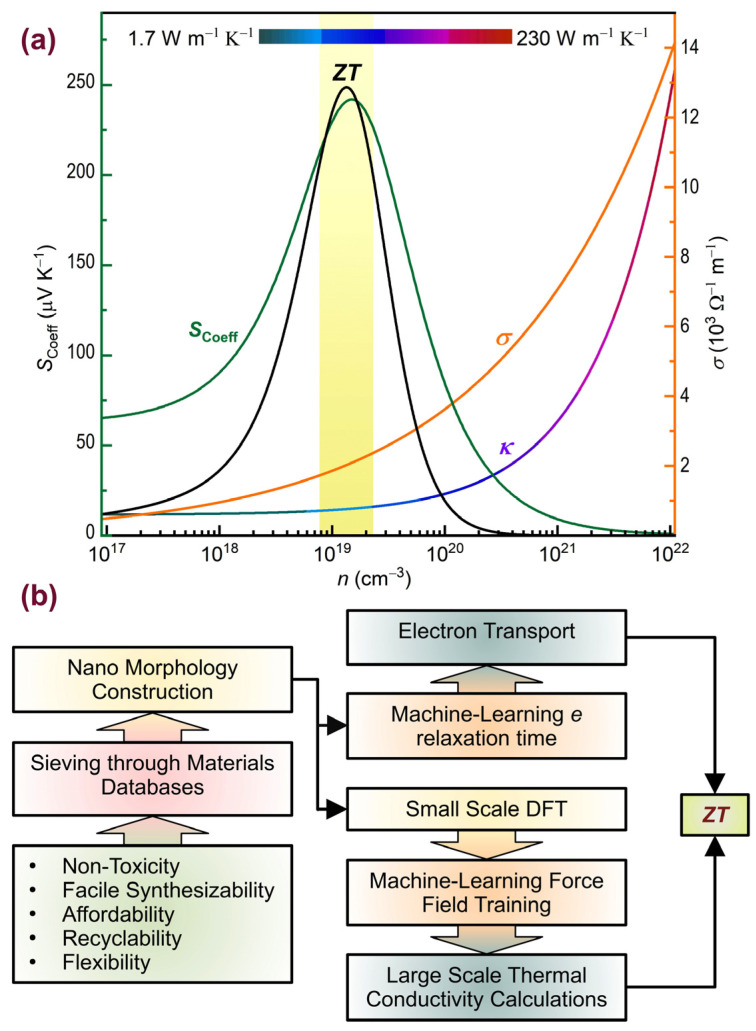
(**a**) The Seebeck coefficient (*S*_Coeff_), electrical conductivity (*σ*), and thermal conductivity (*κ*) of polycrystalline Pb_1−*x*_Sn*_x_*Te as a function of carrier concentration (*n*) at 300 K. *ZT* achieves its peak value within a narrow range of *n*, highlighted in yellow. Varying the temperature shifts these curves in a complex and nontrivial way. (**b**) The proposed workflow for high-throughput nanomaterial screening for finding new *ZT* heights, relying on materials informatics and machine learning techniques. Panel (**a**) adapted from Ref. [12]. Copyright 2020 the authors (CC BY 4.0).

As seen in Figure 7a, the window for carrier concentration (or any other experimentally adjustable parameter) where *ZT* is maximum for any given material is narrow. Traditionally, experimentalists have relied on judicious and assiduous sets of experiments, aggressively engineering candidate materials to fine-tune design parameters for the best TE performance that falls within this narrow range [17,18]. As seen in the literature reviewed before, though there have been numerous independent theoretical investigations, synergetic reliance on computational insight has often been scarce in the rational design of thermoelectric materials. In this regard, theory and experiment have mainly progressed in parallel. The limitation in collaboration stems from a series of computational bottlenecks preventing widescale and dependable computational aid. These bottlenecks were especially severe in calculating the electron relaxation time [12] and the phonon contribution to thermal conductivity [11]. Both quantities are complex to calculate and rely on many approximations, diminishing their predictive relevance to experimental works.

It is important to note that the outstanding works reviewed here have been conducted over the past three decades. Therefore, the computational limitations should be acknowledged accordingly. Moving forward, however, using new data analytic tools that were previously unavailable seems to be indispensable. Noticeably, in the past few years, reliance on data analytic methods in materials discovery has increased exponentially, encompassing many aspects [84,85,86]. Here, we would like to focus on an approach particularly applicable to low-dimensional thermoelectrics: the proliferation of machine learning for calculating the electron relaxation time and the phonon contribution to thermal conductivity.

Calculating the electron relaxation time is challenging because it is influenced by scattering from various mechanisms, including vacancies, interstitial atoms, impurity atoms, grain boundaries, and phonons [87]. Consequently, designing a completely ab initio model to obtain the relaxation time is practically impossible. Therefore, it is frequently approximated as a constant value, disregarding the effects of carrier concentration (doping level) and temperature. When applied to high-throughput surveys, such approximations are bound to result in overly optimistic predictions. However, Hirosawa et al. recently made significant progress in accurately determining the electron relaxation time using machine learning projection trained on experimental data [88]. This technique was successfully applied to Ta_4_SiTe_4_, producing a reasonable match between theory and experiment [89]. At this stage, the transferability of such relaxation time to new materials is yet to be examined.

Using classical molecular dynamics has been computationally the most straightforward and efficient way of estimating the lattice contribution to thermal conductivity, especially for materials with complex morphologies. However, the lack of suitable force fields that produce accurate trajectories for the desired set of elements within an extensive temperature range has always posed a limitation. Recently, machine-learning-based force fields (MLFFs) have offered some hope in this regard [90,91,92]. MLFFs, trained on high-quality density functional data (e.g., as implemented in VASP 6 at https://vasp.at/, accessed 17 July 2024), ensure that the force fields capture the complex interactions between atoms more accurately than empirical potentials. As a result, the calculated thermal conductivity using MLFFs is closer to experimental values [93,94]. Additionally, once trained, MLFFs can perform simulations much faster than ab initio methods for supercells containing tens of thousands of atoms. This speed advantage allows for the simulation of larger systems and longer time scales, allowing the investigation of dilute dopants, low-symmetry structures, and complex interfaces.

The powerful tools described before, combined with impressive materials databases, such as the Materials Project (with approximately 150,000 entries) [95], AtomWork-Adv (containing around 400,000 crystal structures, 500,000 materials properties, and 48,000 phase diagrams) [96], and 2DMatPedia (which includes several thousand two-dimensional materials) [97], can automate high-confidence, high-throughput searches for thermoelectric materials, as schematically shown in Figure 7b. Therefore, it is highly anticipated that future discoveries in thermoelectric nanostructures will depend on a renewed synergy between theory and experiment.

Experimentally, common synthesis routes for one-dimensional materials include electrospinning. This highly efficient top-down method uses electrostatic force to create continuous nanofibres. Moreover, solution-phase methods, like hydrothermal and solvothermal techniques, facilitate chemical reactions under controlled conditions of pH, time, pressure, temperature, and additives. Other techniques include electrochemical deposition methods, such as chemical vapour deposition for semiconductor nanowires, atomic layer deposition for precise thickness control, and confined or oriented template synthesis methods like reactive sputtering [29,98,99]. Future experimental development should address concerns with scalability and uniformity by improving the precise control required over synthesis conditions and reducing the cost and environmental impact by eliminating toxic elements and reagents.

Ensuring the stability of the presented 1D materials at high temperatures and their integration into devices without degrading their thermoelectric properties is a significant challenge. In particular, carbon nanotubes degrade with increasing temperature, primarily through oxidation, leading to carbon dioxide and other gases forming, causing structural damage. An optical absorbance technique investigating the temperature dependence of vertically aligned single-walled carbon nanotube degradation revealed that significant oxidisation starts at *T* = ~400 °C [100]. Multi-walled carbon nanotubes were found to oxidise at even lower temperatures of ~200 °C [101]. Given these limitations, the thermoelectric application of CNTs is limited to temperatures near ambient.

Finally, we would like to draw the readers’ attention to the necessity of a wholesome approach to assessing thermoelectric materials and devices. Though a thermodynamically important factor, the maximum attainable figure of merit *ZT* usually fails to illustrate a complete picture regarding practical application concerns. For instance, economic constraints often set a trade-off between efficiency and cost when expensive elements or techniques are required to increase *ZT*. Efforts have been made to address the incomplete nature of the *ZT* metric by developing an analytical framework based on the cost per watt (USD/W) metric, demonstrating that minimising the cost per watt for thermoelectric materials and systems can sometimes, but not always, align with maximising *ZT* [102].

Furthermore, in some breakthrough thermoelectric materials, the maximum *ZT* occurs within a narrow temperature range. For example, in Bi_0.5_Sb_1.5_Te_3_ [103] and Cu-doped Bi_2_Te_2.7_Se_0.3_ [103], *ZT* peaks around ~1.9 and ~1.2 at temperatures centred at 300 K and 400 K, respectively. However, outside narrow temperature ranges within ±20 K, *ZT* drops rapidly by several folds, leading to reliability issues in real-life applications. Consequently, a comprehensive approach is essential for assessing thermoelectric materials and devices, as the maximum figure of merit *ZT* often fails to address practical application concerns, including economic constraints and temperature-dependent reliability issues.

## 5. Conclusions

This brief review demonstrated how reducing the dimensions of materials can significantly alter their properties due to quantum confinement, enabling the circumvention of the main limitations in thermoelectric technology. One-dimensional nanostructures improve thermoelectric performance by reducing phonon thermal conductivity and enhancing the power factor. Among the materials presented, Si nanowires with reduced thermal conductivity and maintained electrical properties have shown significant *ZT* enhancements, making them promising candidates for thermoelectric devices when integrated with other Si-based semiconducting components. However, carbon nanotubes offer flexibility and high specific energy, broadening the use cases for thermoelectric materials. The thermal conductivity in carbon nanotubes can be reduced to competitive values of ~10 W m^−1^ K^−1^ by introducing defects and preparing the nanotubes in the bundle, fibre, film, and bucky-paper forms. Combined with one of the highest power factors among one-dimensional structures, the *ZT* value for CNT-based TE devices can reach up to 0.42 at 300 K. Building on these significant achievements to date and the latest computational advancements, including machine learning techniques for determining the electron relaxation time and machine-learning-based force fields for estimating thermal conductivity, the future of thermoelectric research is poised to increasingly depend on theory-guided experiments.

## Data Availability

Data sharing is not applicable to this article, as no new data were created in this study. All data reported here can be traced back to the cited literature.
